# Hepatobiliary Brucellosis: Brucella Bacteremia Presenting with Refractory Hepatobiliary Infection

**DOI:** 10.1155/2023/5513052

**Published:** 2023-11-11

**Authors:** Dania El Hallak, Mae Al Habbal, Wael Hassan Zorkot

**Affiliations:** ^1^Beirut Arab University, Beirut, Lebanon; ^2^Lebanese University, Lebanon

## Abstract

Brucellosis often presents with common and nonspecific symptoms such as fever, malaise, and arthralgia but can also involve primary organs. Intra-abdominal involvement is rare. We report a case of hepatobiliary brucellosis presenting as a refractory hepatobiliary infection in a healthy young adult with no underlying rheumatologic disease or history of exposure to risk factors. Detection of Brucella in the blood led to a shift in the patient's management and consequently her recovery.

## 1. Background

Brucellosis has a total of nine other names, most prominently the Mediterranean and Malta fevers, and is caused by small aerobic, gram-negative coccobacillus bacteria [1, 2]. It causes a multisystem disease that has a broad spectrum of clinical manifestations such as fever, malaise, night sweats, arthralgia, abdominal pain, and weight loss [3, 4]. According to studies, in about 30% of cases, brucellosis can involve one or more focal sites, including osteoarticular, genitourinary, neurological, cardiovascular, ocular, and intra-abdominal sites [5, 6]. We report a case of a previously healthy female patient presenting with an atypical picture of acute cholecystitis complicated by sepsis and disseminated intravascular coagulation (DIC) and associated with a late finding of Brucella bacteremia.

## 2. Case Presentation

This is a case of a 29-year-old, G3P3A0 female patient, known to be previously healthy and has no history of rheumatological disease, presenting to a tertiary care center in Beirut, with right upper quadrant pain associated with fever and chills that started 12 days ago. The patient is a housewife who likes to drink milk and have dairy products freshly obtained from her small farm. There is no history of recent travel or contact with someone who was travelling abroad. She sought medical advice once and was prescribed analgesics, paracetamol, only. One day prior to presentation, she sought surgical consultation and had an ultrasound done in an outpatient setting, which showed mild ascites and a distended gallbladder suggestive of reactive cholecystitis. An elective laparoscopic cholecystectomy was scheduled for the following week. However, a few days later, the patient started to become lethargic with failure to tolerate PO intake, so she was rushed back to the hospital. Upon presentation, the patient was somnolent and jaundiced. Her vital signs were unstable, hypotensive (SBP 70 mmHg), and tachycardic, but oxygen saturation was normal (97-98%). She had an estimated BMI of 29.

As listed in Table 1, laboratory studies showed thrombocytopenia, anemia, and neutropenia. Her pT and pTT levels were prolonged, and fibrinogen was consumed, suggesting ongoing disseminated intravascular coagulopathy (DIC).

Her chest X-ray showed an enlarged heart, slightly congested lungs with bilateral mild pleural effusion, and bibasilar reticulation/atelectatic changes. Blood cultures were taken. Four liters of normal saline as IV hydration were given, and FFPs 1U q 8 hours and 1 pool of platelets were transfused. Tazocin 4.5 g IVD q 6 hours was started, in addition to metronidazole 500 mg IVD q 8 hours. However, no improvement was seen, so vasopressors were given (Levophed 8 mg) with a target blood pressure > 110 mmHg. After her vitals stabilized, the patient underwent an urgent laparoscopic cholecystectomy with intraoperative cholangiography (IOC), without a prior MRCP, due to her condition. No photos were taken during the surgery. Extubating the patient failed as she developed ARDS, complicated by alveolar hemorrhage, so she was reintubated and transferred to the intensive care unit (ICU) immediately after the operation. In ICU, she was sedated with Dormicum and fentanyl, received nutritional feeding via a nasogastric tube at a rate of 60 cc/hr, and was given FFPs every 12 hours along with multiple blood transfusions. Blood cultures that were taken on admission on 23.11.2022 turned negative after the regular incubation period of 5 days.

During her ICU stay, our patient developed septic shock, so another set of blood cultures were taken and followed by initiating empiric broad-spectrum antibiotics. Meropenem 1 gram every 8 hours and Tavanic 750 mg every 24 hours were started, in addition to colistin that was given for 2 days for persistent fever. However, no clinical or laboratory improvement was observed. One week later, blood cultures detected Brucella species, and Brucella tube agglutination titer was found to be higher than 1/5120. Laboratory workup done will be summarized in Table 2. Doxycycline 100 mg every 12 hours and rifampicin 600 mg once daily per IV were started immediately.

After initiation of anti-Brucella treatment, rapid clinical and laboratory improvement was perceived as platelets and WBC count were elevated. Repeated investigations such as chest X-rays and blood studies were found unremarkable. TTE was also done and showed normal heart function, with no signs of hypokinesia and no vegetations present. The patient was transferred to the regular floor a few days later and was discharged on oral doxycycline and rifampicin.

## 3. Discussion

Brucellosis can be transmitted by consuming unpasteurized food or coming into direct contact with diseased tissues or fluids of infected animals, as cited by the World Organization for Animal Health [7]. Brucellosis is still endemic in the Middle East, according to the World Health Organization [8]. It is considered a serious public health concern in the eastern, southern, and northern regions of Lebanon [8].

Fever is the most common presentation of brucellosis, which is a well-known cause of undulant fever. Night sweats, malaise, anorexia, arthralgia, fatigue, and weight loss are other common and nonspecific symptoms. Presentation may be abrupt or slow over days to weeks. Physical findings are usually absent except for fever; lymphadenopathy and mildly enlarged liver and spleen may occasionally be found [9]. Studies have shown that in about 30% of cases, Brucella disseminates via the bloodstream or lymphatics and affects primary organs causing localized disease [9, 10]. Osteoarticular disease, such as peripheral arthritis, sacroiliitis, and spondylitis, is the most common form of this disease manifestation [11]. Intra-abdominal involvement, which may include hepatic or splenic abscess, cholecystitis, ileitis, and colitis, is rare [5]. The etiology of cholecystitis related to brucellosis is unknown. Brucella may enter the gallbladder after causing bacteremia, and prolonged latent infection may result in the production of gallstones [12].

Blood, body fluids, and tissue cultures are used to establish the definitive diagnosis of brucellosis [13]. Tetracyclines, aminoglycosides, and rifampicin are antibiotics with activity in the acidic intracellular environment and are used for eradication of Brucella bacteria [14]. Due to the high likelihood of relapse with monotherapy, the use of combination therapy (gentamicin/streptomycin or rifampicin with doxycycline) for a lengthy period of time, of about six weeks, is advised [15].

In this case report, the patient had intermittent fever, chills, and right upper quadrant pain for 2 weeks, in addition to jaundice, and presented with features of DIC and hemodynamic instability. Past medical history is negative for previous hospitalizations and exposure to risk factors, and she has no underlying rheumatologic disease [16]. US done before admission showed acute cholecystitis; however, based on her presentation, suppurative cholangitis is also suspected.

The patient was admitted for urgent laparoscopic cholecystectomy that failed to alleviate her clinical deterioration. After the operation, she developed ARDS and was admitted to the ICU where she developed septic shock a few days later, so she was started on broad-spectrum antibiotics (carbapenems and vancomycin) but showed no response. Blood cultures that were withdrawn upon presentation to the ER were obtained and grew gram-negative coccobacilli identified as Brucella, so doxycycline and rifampicin were started, and the patient showed rapid clinical and laboratory improvement.

The resolution of sepsis and clinical improvement after starting anti-Brucella treatment identifies an association between Brucella bacteremia and refractory hepatobiliary disease in our patient that led to septic shock and DIC due to the delay in diagnosis and proper management, as it failed to respond to the usual first-line interventions such as surgery and broad-spectrum antibiotics.

## 4. Methodology

This section describes the methods used by our research team to collect and analyze this case report. This is a single-patient retrospective study that is conducted in a single institution. Data was gathered by the corresponding authors using different strategies that could help view the patient's profile and history adequately and thoroughly. Data collection process started by interviewing the general surgery residents who admitted the patient initially, and then the internal medicine residents who managed the in-hospital care of the patient during her hospital stay. Secondary sources like archival records on the hospital electronic information system were used to gather all laboratory investigation results and progress note documentation. Direct observation by the corresponding authors was conducted throughout the study as a source of evidence to validate the information claimed by the system and the professionals who were interviewed. All data gathered was also evaluated and examined by an infectious disease specialist, who sorted the history and events in chronological order to analyze the case and confirm the acclaimed diagnosis and validate the correctness of management done to the patient.

## 5. Limitations

This study faced some limitations arising from the methods that were used to collect all necessary data. Interviews hold an element of bias that could arise from a poor choice of questions, communication failure between the interviewer and the recipient, subjective judgement of the case, incomplete recollection of information, and reflectivity. Direct observation can include an element of selectivity. Documentation of progress notes written by residents can reflect author bias and reporting bias.

## 6. Conclusion

This is a rare case of Brucella bacteremia presenting as a refractory hepatobiliary infection with a complicated clinical course, in a patient with no history of rheumatologic disease or other risk factors. Brucellosis may rarely reach intra-abdominal organs to cause cholecystitis or cholangitis, as in our case, which increases morbidity. This case highlights the need to keep brucellosis at the top of the differential diagnosis in patients presenting with refractory hepatobiliary infections in endemic areas.

## Figures and Tables

**Table 1:** Initial laboratory findings.

Variables	Laboratory studies on day 1 (24.11.2022)	Reference range
Hemoglobin	7.3	11.8-16 g/dL
Hematocrit	22.9	36-47%
Platelets	55	150-400 10^3/ul
White blood cell count	1.8	4-11 10^3/ul
INR	1.86	0.8-1.2
Prothrombin time	24.1	11-13 sec
APTT, plasma	79.3	25-40 sec
LDH	1484	135-225 U/L
D-dimer	2.58	<0.5 UG/ML
Fibrinogen	43	200-400 mg/dL
Sodium	142	130-145 mmol/L
Potassium	2.93	3.5-5.4 mmol/L
Chloride	83	93-110 mmol/L
Phosphate	2.0	2.4-4.5 mg/dL
Bicarbonate	20	20-30 mmol/L
Blood urea nitrogen	9	5-25 mg/dL
Creatinine	0.54	0.5-1 mg/dL
Alanine transaminase	142	0-50 UL
Aspartate transaminase	430	0-50 UL
Gamma-glutamyl transferase	101	5-36 UL
Total bilirubin	4.5	0-1.5 mg/dL
CRP	4.78	<0.3 mg/dL

**Table 2:** Laboratory studies in the intensive care unit (ICU).

Variables	Laboratory studies on day 10 (3.12.2023)	Reference range
Hemoglobin	10.8	11.8-16 g/dL
Hematocrit	32.3	36-47%
Platelets	105	150-400 10^3/ul
White blood cell count	4.1	4-11 10^3/ul
INR	1.01	0.8-1.2
Prothrombin time	13.6	11-13 sec
APTT, plasma	32.4	25-40 sec
Sodium	136	130-145 mmol/L
Potassium	2.84	3.5-5.4 mmol/L
Chloride	102	93-110 mmol/L
Phosphate	2.5	2.4-4.5 mg/dL
Bicarbonate	25	20-30 mmol/L
Blood urea nitrogen	12	5-25 mg/dL
Creatinine	0.27	0.5-1 mg/dL
Alanine transaminase	31	0-50 UL
Aspartate transaminase	36	0-50 UL
Gamma-glutamyl transferase	427	5-36 UL
CRP	8.55	<0.3 mg/dL
Brucella titer	1 : 5120	<1 : 320

## Data Availability

Data is available from the first authors upon request.
